# Strengthening influenza surveillance capacity in the Eastern Mediterranean Region: Nearly two decades of direct support from the United States Centers for Disease Control and Prevention

**DOI:** 10.1111/irv.13220

**Published:** 2023-11-05

**Authors:** Kinda Zureick, Margaret McCarron, Patrick Dawson, Jamie K. Davis, John Barnes, David Wentworth, Eduardo Azziz‐Baumgartner

**Affiliations:** ^1^ Influenza Division US Centers for Disease Control and Prevention Atlanta Georgia USA

**Keywords:** capacity building, Eastern Mediterranean Region, human, influenza, public health surveillance, sentinel surveillance

## Abstract

Since 2004, the US Centers for Disease Control and Prevention (CDC) Influenza Division (ID) has supported seven countries in the Eastern Mediterranean region and the World Health Organization Regional Office for the Eastern Mediterranean to establish and strengthen influenza surveillance. The substantial growth of influenza surveillance capacities in the region demonstrates a commitment by governments to strengthen national programs and contribute to global surveillance. The full value of surveillance data is in its use to guide local public health decisions. CDC ID remains committed to supporting the region and supporting partners to translate surveillance data into policies and programs effectively.

## 1 THE IMPORTANCE OF NATIONAL CAPACITIES FOR INFLUENZA SURVEILLANCE

The ever‐evolving influenza viruses are an ongoing and significant threat to public health, including those circulating in animal reservoirs that could generate a pandemic. Each year, epidemic type A and B influenza viruses are estimated to infect 1 billion people, cause severe disease in 3–5 million, and lead to death in 290,000–650,000.^1^ Although there are safe and effective vaccines and antiviral treatments for influenza A and B viruses, keeping vaccine policy recommendations and vaccine antigen composition up to date relies on a thorough understanding of the specific viruses currently co‐circulating in various populations.

The World Health Organization's (WHO) Global Influenza Surveillance and Response System (GISRS) enables global monitoring of circulating influenza virus strains to improve the selection and development of candidate vaccine viruses for human epidemic and pandemic influenza vaccines.^2^ Meaningful participation in the GISRS network depends on leveraging national capacities, like the ability to detect influenza virus infections using laboratory diagnostic methods, monitor the spread of disease across the population, report associated virologic and epidemiologic information on time, and share a subset of representative specimens with WHO Collaborating Centers (WHO‐CCs). These data, additional genetic and antigenic data, and candidate vaccine viruses derived from specimens submitted by national influenza reference laboratories inform biannual global recommendations for vaccine antigen composition.

Robust national influenza surveillance benefits decision‐making at the national level and contributes to global health priorities. Many influenza surveillance systems are fundamentally based on laboratory capacities, where virus detection provides information on the temporal patterns of virus transmission and circulating strains. Epidemiologic data, when coupled with virologic data, provide insight into populations that are most vulnerable to influenza‐associated illnesses and death. When optimized, surveillance data provide timely awareness of the impact of influenza viruses that enables health authorities to track epidemic influenza activity, assess the severity of disease, investigate outbreaks, respond to pandemic threats (i.e., novel virus outbreaks), and implement data‐driven mitigation policies and programs, like targeted vaccinations for the most vulnerable populations, to protect their communities.

## 2 CDC'S SUPPORT OVER THE YEARS

The US Centers for Disease Control and Prevention (CDC) Influenza Division (ID) has extended and expanded direct financial and technical support to partners globally since 2004.^3^, ^4^, ^5^ Seven countries within the WHO Eastern Mediterranean Region (EMR)—Afghanistan, Egypt, Tunisia, Lebanon, Morocco, Pakistan, and Somalia—and the WHO Regional Office for the Eastern Mediterranean (WHO EMRO) have received support from CDC ID to build and strengthen national influenza surveillance and pandemic preparedness. To date, nearly $20 million has been dedicated to support surveillance activities in these countries, and $22 million has been dedicated to support WHO regional office initiatives. The International Reagent Resource (IRR, formerly named the Influenza Reagent Resource) provides free‐of‐cost reagents, test kits, and information for the detection of influenza to 21 GISRS laboratories (as of June 2023) in EMR. Since the onset of the COVID‐19 pandemic, IRR has distributed 600 kits, supporting 300,000 tests for influenza and severe acute respiratory syndrome coronavirus 2 (SARS‐CoV‐2) in the region, in addition to the continued distribution of influenza subtype and lineage test kits.

In collaboration with WHO EMRO, CDC ID annually supports regional trainings and workshops and provides technical assistance to improve the quality and timeliness of influenza surveillance. These initiatives have broad reach and are not limited to countries receiving direct ID funding. For instance, CDC ID staff have facilitated multi‐country epidemiologic‐focused workshops on data management and analytics, burden of disease estimation, pandemic preparedness planning, and evaluation of surveillance systems. They have also provided substantial support to WHO‐sponsored courses and have trained national influenza laboratories in EMR on molecular detection via reverse transcription polymerase chain reaction (RT‐PCR) and genomic sequencing (most recently via Sanger sequencing in 2018 and next‐generation sequencing in 2022).

In addition to bolstering surveillance capacity, CDC ID has worked with partners in EMR to catalyze the expansion of influenza vaccination programs and increase access to influenza vaccines. Countries in EMR have received direct funding and technical assistance from CDC and additional support through a CDC‐Task Force for Global Health (TFGH) collaboration. Through these initiatives, technical support was provided to conduct surveys and focus groups to understand vaccine demand, conduct vaccine communication campaigns, convene regional forums to identify evidence gaps and needs for vaccine program development and expansion, and receive limited vaccine donations via the Partnership for Influenza Vaccine Introduction at TFGH. This portfolio of work offers a glimpse into the broader impact of using surveillance data to inform national programmatic and policy decisions.

## 3 EVIDENCE OF EXPANDED REGIONAL CAPACITIES

During nearly two decades of direct support to EMR, there has been substantial growth in influenza surveillance programs in the region. Sentinel surveillance has been established in 19 countries. Reporting of virologic surveillance data to WHO FluNet or Eastern Mediterranean Flu (EMFLU) Network, the global and regional influenza surveillance reporting platforms, respectively, have increased from 4 countries in 2004 to 18 in 2023.^6^ Mechanisms are in place in at least 18 countries to routinely test and report virologic data year‐round or during seasonal periods of increased respiratory virus circulation.

The number of WHO‐designated National Influenza Centres (NICs) in the region increased from 13 to 18 between 2011 and 2023.^2^, ^7^ NIC designation signifies that a national laboratory meets competency standards for virus detection, typically using real‐time RT‐PCR. Further, by differentiating type A and B viruses and often, subtyping influenza A (e.g., H1N1pdm09) virus or lineage testing influenza B (e.g., B/Victoria or B/Yamagata), national laboratories are better equipped to regularly identify representative specimens to share with WHO‐CCs for more detailed characterization and to rapidly identify and send viruses that do not normally infect humans to the WHO‐CCs of GISRS. Since 2004, 25 candidate vaccine viruses for human influenza vaccine have originated from national laboratories in EMR, of which 4 were selected for manufacturing or preparedness.^8^

Through both published and unpublished time‐series analyses, countries in the region have demonstrated a more thorough understanding of influenza circulation patterns and seasonal trends, and some countries have determined epidemic thresholds to better guide their seasonal response to influenza spread.^9^, ^10^, ^11^, ^12^ In 2019, representatives from 14 ministries of health in the region received training in and began a systematic evaluation of their influenza surveillance systems based on CDC's guidelines for evaluating public health surveillance systems and used the findings for influenza surveillance program decision‐making.^13^, ^14^

The substantial improvements in surveillance capacities supported by CDC ID in collaboration with WHO EMRO demonstrate commitment by national governments in EMR to prioritize programs for influenza surveillance, response, and preparedness and contribute data and specimens for consideration in global influenza vaccine composition recommendations. These capacities have served as the basis for the diagnosis and investigation of other infectious diseases, like Middle East respiratory syndrome coronavirus (MERS‐CoV), Ebola, and SARS‐CoV‐2.^4^, ^15^

## 4 FUTURE OPPORTUNITIES

Generating influenza surveillance data is crucial in understanding the dynamics of rapidly evolving influenza viruses. However, merely collecting data without translating it into actionable insights (i.e., using it to inform local public health decision‐making) diminishes the full value of surveillance efforts. Thus, health ministries with influenza surveillance systems might consider routinely conducting analyses to detect the start or end of seasonal activity and peak or off‐season activity, characterize the severity of seasonal epidemics, identify populations most impacted by influenza, and identify predominantly circulating influenza strains. When regularly examined, this information can help inform the optimal timing of influenza vaccination or other intervention campaigns and support targeted messaging, such as promoting influenza vaccination among priority populations and disseminating updated treatment guidelines to clinical networks. It can also inform healthcare resource allocation if jurisdictions within a country experience particularly severe epidemics or alert stakeholders to potential outbreaks (i.e., unusual clusters of cases or infection with an influenza virus not normally circulating in humans) that warrant further investigation. Furthermore, to better understand the true national impact of influenza viruses, health ministries can consider in‐depth evaluations that build upon routine surveillance data, like estimating numbers of averted illnesses or the burden of disease and determining the cost‐effectiveness of investments in influenza vaccines and antivirals, which taken together can help demonstrate the value proposition of expanding influenza prevention and mitigation measures.

Bridging the gap between influenza data generation and effective consumption requires a systemic shift in institutional frameworks to foster a culture of using surveillance data to inform routine and timely evidence‐based decision‐making. As countries consider sustainably expanding influenza surveillance platforms to monitor for other priority respiratory viruses (e.g., SARS‐CoV‐2 or respiratory syncytial virus), they should critically assess and align their surveillance priorities so that the data produced from surveillance systems can be used to their full potential. Enhancing the skills necessary to translate data into policy and practice will undoubtedly aid health ministries in responding to the annual circulation of influenza viruses. CDC ID remains committed to supporting influenza surveillance capacity building in EMR to bridge the gap between data collection and use for public health action.

## AUTHOR CONTRIBUTIONS

**Kinda Zureick:** Conceptualization (lead); data curation (equal); writing—original draft (lead); writing—review and editing (lead). **Margaret McCarron:** Conceptualization (equal); data curation (equal); writing—original draft (equal); writing—review and editing (equal). **Patrick Dawson:** Conceptualization (equal); data curation (equal); writing—original draft (equal); writing—review and editing (equal). **Jamie Kristy Davis:** Conceptualization (supporting); data curation (equal); writing—original draft (equal); writing—review and editing (equal). **John Barnes:** Conceptualization (equal); data curation (equal); writing—original draft (equal); writing—review and editing (equal). **David Wentworth:** Conceptualization (equal); data curation (equal); writing—original draft (equal); writing—review and editing (equal). **Eduardo Azziz‐Baumgartner:** Conceptualization (equal); data curation (equal); writing—original draft (equal); writing—review and editing (equal).

## CONFLICT OF INTEREST STATEMENT

The authors declare no conflict of interest declared.

### PEER REVIEW

The peer review history for this article is available at https://www.webofscience.com/api/gateway/wos/peer-review/10.1111/irv.13220.

## Data Availability

The data that support the findings of this study are available on request from the corresponding author. The data are not publicly available due to privacy or ethical restrictions.
